# Plant-derived extracellular vesicles as a novel tumor-targeting delivery system for cancer treatment

**DOI:** 10.3389/fcell.2025.1589550

**Published:** 2025-06-24

**Authors:** Hanjin Wu, Mengya Shi, Liwei Meng, Jie Qiu, Yuancong Jiang, Da Qian, Fengqing Shen

**Affiliations:** ^1^ Department of Breast and Thyroid Surgery, Shaoxing People’s Hospital, Shaoxing, Zhejiang, China; ^2^ Department of Cadre Healthcare, Shaoxing People’s Hospital, Shaoxing, Zhejiang, China; ^3^ Department of Burn and Plastic Surgery-Hand Surgery, Changshu Hospital Affiliated to Soochow University, Changshu No. 1 People’s Hospital, Changshu, Jiangsu, China

**Keywords:** plant-derived extracellular vesicles, solid tumors, tumor microenvironment, antitumor therapy, precision medicine

## Abstract

Extracellular vesicles (EVs) are vital mediators of intercellular communication, helping to transfer bioactive molecules to target cells and demonstrating significant potential in antitumor therapy. Currently, EVs are primarily utilized in clinical applications such as biomarker discovery, cell-free therapeutic agents, drug delivery systems, pharmacokinetic studies, and cancer vaccines. Plant-derived EVs (P-EVs) contain a range of lipids, proteins, nucleic acids, and other metabolite cargos, and it is possible to extract them from various plant tissues, including juice, flesh, and roots. These vesicles perform multiple biological functions, including modulating cellular restructuring, enhancing plant immunity, and defending against pathogens. P-EVs have also been investigated in various clinical trials due to their promising therapeutic properties. In the context of precision medicine, selectively inhibiting solid tumor growth while preserving the viability of normal human cells remains a primary objective of cancer therapy. However, the tumor microenvironment (TME) supports tumor progression through the facilitation of immune evasion, supplying nutrients, and promoting invasive growth, metastatic processes, and treatment resistance. Consequently, the development of novel antitumor agents is essential. Owing to their inherent therapeutic properties and potential as treatment vectors, natural P-EVs represent a promising biocompatible platform for targeted solid tumor therapy. These vesicles may contribute to remodeling the TME and enhancing antitumor immunity, offering innovative avenues for cancer treatment and improved human health.

## Background

Cancer remains one of the most prominent global causes of mortality. The International Agency for Research on Cancer estimates there were 20 million new cancer cases and 10 million cancer-related deaths recorded in 2024. Lung cancer (12.4%) has surpassed breast cancer (11.6%) as the most frequent cancer diagnosis, in addition to being the largest driver of cancer-associated mortality (18.7%), followed by colorectal cancer (9.3%) (Siegel et al., 2024; Qian et al., 2025). Therefore, the need for effective antitumor therapies remains a critical challenge. Conventional therapies for cancer, including surgery, chemotherapy, radiotherapy, and targeted therapy, are widely used (Anand et al., 2023; Zhan et al., 2023). Although these strategies effectively eliminate cancer cells, they are linked to significant adverse effects, such as delayed recovery and loss of physiological function following surgery, as well as organ toxicity and myelosuppression induced by chemotherapy, all of which adversely impact patients’ quality of life (Liu Y. Q. et al., 2021; Ghanbar and Suresh, 2024).

The emergence of personalized medicine has marked a turning point in cancer treatment, offering a more tailored approach to patient care. However, tumor heterogeneity and the complex compensatory mechanisms of the TME contribute to drug resistance and reduced treatment efficacy, leading to unsatisfactory outcomes in many clinical trials of precision medicine (Joo et al., 2020). Consequently, researchers continue to seek alternative therapeutic strategies that selectively target tumor cells while minimizing harm to healthy tissues, ultimately aiming to reduce treatment-related toxicity, provide patients with a better quality of life, and extend survival.

**FIGURE 1:**
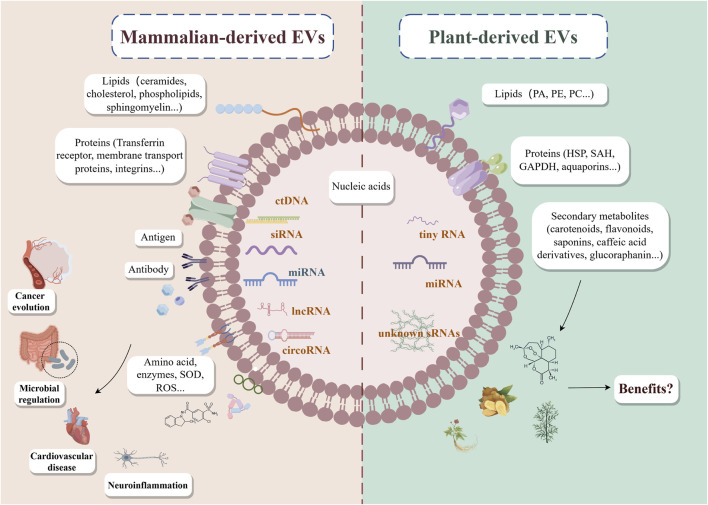
Current status of mammalian-derived EVs and plant-derived EVs. EVs, extracellular vehicles; PA, phosphatidic acid; PE, phosphatidylethanolamine; PC, phosphatidylcholine; SOD, superoxide dismutase; ROS, reactive oxygen; HSP, heat shock protein 70; SAH, S-adenosyl-homocysteinase; GAPDH, glyceraldehyde 3-phosphate dehydrogenase; ctDNA, circulating tumor DNA; siRNA, small interfering RNA; miRNA, microRNA; lncRNA, long non-coding RNA; circRNA, circular RNA; sRNAs, this refers to other undiscovered small RNAs.

Exosomes, with an average diameter of approximately 100 nm, are nanoscale vesicles secreted by nearly almost all cells under both pathological and physiological conditions (Kalluri and LeBleu, 2020). These vesicles facilitate the transfer of diverse biomolecules, including mRNAs, miRNAs, proteins, lipids, and metabolites, from donor to recipient cells, influencing immunity, viral pathogenicity, pregnancy, cardiovascular diseases, neurological disorders, and cancer progression (He et al., 2021; Qiu et al., 2024) (Figure 1). Recently, research on EVs has predominantly focused on mammalian cell-derived exosomes. For instance, EVs from mesenchymal stem cells (MSC-EVs) have been explored for their potential in drug delivery, cancer immunotherapy, and regenerative medicine (Weng et al., 2021; Keshtkar et al., 2018). Additionally, EVs derived from cancer-associated fibroblasts (CAF-EVs) play central roles in inducing metabolic reprogramming and mediating immune suppression within the TME (Li et al., 2021). Despite their promising therapeutic potential, mammalian-derived exosomes pose certain risks, including immune rejection and the potential transmission of harmful substances such as tumor-associated molecules and infectious agents, coupled with the complexity of their own production and their scarcity, which present major challenges for cell-free therapy (Hanayama, 2021). In recent years, growing interest has emerged in the utilization of EVs derived from plants and traditional Chinese medicine for cancer treatment.

Plant-derived EVs (P-EVs) possess multiple bioactive cargos akin to those found in mammalian-derived EVs. Increasing evidence suggests that P-EVs contain bioactive molecules with diverse functions. For example, exosome-like nanoparticles (ELNs) from ginger rhizomes can readily suppress the activation of the NLRP3 inflammasome, suggesting their potential as novel agents to suppress inflammasome assembly and activation (Chen et al., 2019). Similarly, the plant-derived compound curcumin modulates Nrf2, MAPK, and NF-κB signaling, exhibiting antioxidant properties, mitigating chemotherapy and radiotherapy-related side effects, and scavenging free radicals (Liu Y. Q. et al., 2021). Furthermore, plant-based compounds such as flavonoids, quercetin, and coumarins have demonstrated anti-inflammatory, antioxidant, immunomodulatory, and anticancer properties (Naksuriya et al., 2022; Zhao et al., 2019). While both P-EVs and plant extracts can provide therapeutic potential, P-EVs possess distinct advantages, including their encapsulation and delivery of bioactive molecules to recipient cells, enhancing bioavailability and therapeutic efficacy. As a promising candidate for solid tumor treatment, P-EVs may improve therapeutic outcomes by influencing tumor cell proliferation, apoptosis, metastasis, and TME homeostasis (Rome, 2019; Xu et al., 2023). Although P-EV-based therapies remain in the research phase, encouraging preclinical results have been achieved, and clinical trials are being conducted for many cancers, signaling a potential shift toward natural medicine-based cancer treatment.

This review offers a comprehensive overview of the plant sources of P-EVs, summarizes preclinical studies investigating their role in oncology, explores their impact on the TME. The objective is to highlight the promising future of P-EVs as novel therapeutic agents in oncology.

## Composition and isolation of P-EVs

Over the past decade, EVs derived from plant cells have been established as biogenically and morphologically similar to their mammalian counterparts (An et al., 2007). Various terms, including P-EVs, edible plant-derived nanovesicles, plant-derived nanovesicles, and plant exosome-like nanovesicles, have been used to describe these nano-to micro-sized vesicles (50–1,000 nm) (Cui et al., 2020). P-EVs encapsulate a variety of bioactive molecules and function as mediators of communication between cells. For instance, studies indicate that P-EVs are rich in enzymes essential for the remodeling of the cell wall and proteins with antibacterial properties (Woith et al., 2019; Liu G. et al., 2021). Additionally, P-EVs can transport small non-coding RNAs among cells, influencing gene regulation, proliferation, and differentiation (Urzì et al., 2021). Evidence suggests that P-EVs exhibit stable intrinsic therapeutic activity, can be internalized by human cells, and influence cellular processes. Owing to their natural origin and minimal toxicity, P-EVs have emerged as attractive tools for cancer treatment.

To date, four principal compounds have been detected in P-EVs: proteins, nucleic acids, lipids, and secondary metabolites, with all of these playing crucial roles in the stability and function of these vesicles (Cai et al., 2021; Martínez-Ballesta et al., 2018).

## Lipids

Previous studies have identified phosphatidic acid (PA), phosphatidylethanolamine (PE), and phosphatidylcholine (PC) as the major lipid constituents of P-EVs (Karamanidou and Tsouknidas, 2021). In contrast, mammalian-derived EVs predominantly consist of ceramides, cholesterol, phospholipids, and sphingomyelin (Donoso-Quezada et al., 2021). Differences in lipid composition influence the uptake of these vesicles by gut microbiota. For example, ginger-derived EVs enriched in PA exhibit preferential absorption by *Lactobacillus rhamnosus*, whereas grapefruit-derived EVs containing higher levels of PC are taken up predominantly by *Ruminococcaceae* (Teng et al., 2018). This suggests that specific lipid components may serve as molecular cues guiding preferential uptake by distinct intestinal microbiota, highlighting the potential of lipid-based targeting strategies for gut microbiota modulation in anti-tumor therapy. However, the precise mechanisms governing the absorption and internalization of P-EVs by target cells remain poorly understood, necessitating extensive lipidomic studies to elucidate the roles of these lipid components.

## Proteins

Proteins within EVs facilitate host cell uptake and act as carriers of critical genetic information. While proteins in P-EVs play essential roles in plant physiology, their overall content is lower than that of mammalian-derived EVs. Pinedo et al. identified three conserved protein families in P-EVs: heat shock protein 70 (HSP-70), S-adenosyl-homocysteinase, and GAPDH in apoplast washing fluid (Pinedo et al., 2021). Notably, HSP-70 is linked to tumor invasion and treatment resistance in human cancers (Shevtsov et al., 2018) suggesting a potential but unexplored connection between P-EV proteins and their therapeutic effects. Membrane proteins in P-EVs also enhance their uptake by mammalian cells. For example, aquaporins on the surface of broccoli-derived P-EVs contribute to membrane stability and facilitate cellular absorption (Martínez-Ballesta et al., 2018). Additionally, cytoplasmic proteins play roles in cell wall remodeling and pathogen defense (De Palma et al., 2020). Although various studies have characterized P-EV protein compositions, many of their therapeutic properties remain unknown due to challenges in protein extraction. Further proteomic analyses are required to fully uncover their biological functions.

## Nucleic acids

P-EVs contain nucleic acids, such as microRNAs (miRNAs), which regulate gene expression and receptor cell function (López de Las Hazas et al., 2023). Plant-derived miRNAs have demonstrated potential in preventing and treating various human diseases, including cancer (Yi et al., 2025), cardiovascular disorders (Hou et al., 2018), neurodegenerative diseases (Saiyed et al., 2022), and diabetes (Bajaj et al., 2025). For instance, Chin et al. reported that plant miRNA miR159 undergoes natural 2′-O-methylation at its 3′-end, which protects it from degradation and enhances its stability. A synthetic miR159 mimic was found to suppress breast cancer cell proliferation in mice via targeting TCF7, a WNT signaling transcription factor, resulting in reduced MYC protein levels while sparing normal breast epithelial cells (Chin et al., 2016). This study offers evidence that plant-derived miRNAs can inhibit tumor growth in mammalian models. Additionally, Teng et al. identified small RNAs and miRNAs in P-EVs that regulate gut microbiota composition and its metabolites, suppress inflammation, and mediate microbiota-host immune system crosstalk (Teng et al., 2018). Furthermore, Baldrich et al. discovered highly enriched “tiny RNAs” (10–17 nucleotides) in *Arabidopsis thaliana*-derived EVs, although their functions remain unclear (Baldrich et al., 2019). Advances in bioinformatics and transcriptomics are expected to reveal novel pharmacological and therapeutic roles of nucleic acids in P-EVs. Increasingly, research suggests that nucleic acids within P-EVs may target mammalian genes associated with inflammation and cancer, underscoring their potential as therapeutic agents.

## Metabolites

Plant-derived secondary metabolites, including carotenoids, flavonoids, saponins, curcuminoids, and caffeic acid derivatives, possess significant biochemical activity and therapeutic potential (Woith et al., 2021; Bailly and Vergoten, 2020). These metabolites may be encapsulated within P-EVs, contributing to their intrinsic therapeutic properties. For instance, the active compounds 6-gingerol and 6-shogaol, found in ginger, can be loaded into P-EVs and exhibit anti-inflammatory and anti-cancer activities (Bischoff-Kont and Fürst, 2021; Sharma et al., 2023; Karatay et al., 2020). Zeng et al. demonstrated that naringenin, a flavonoid present in grapefruit-derived EVs, has therapeutic potential in treating inflammation-related conditions, including sepsis, fulminant hepatitis, fibrosis, and cancer (Zeng et al., 2018). Table 1 provides a brief overview of the sources of some secondary metabolites or active constituents and their current therapeutic effects. However, not all plant-derived metabolites are incorporated into P-EVs. This variability may stem from differences in the lipid and membrane protein compositions of P-EVs across plant species, as well as physicochemical properties such as lipid or water solubility and molecular density. The mechanisms governing the selective packaging of metabolites into P-EVs remain unclear, highlighting the need for further research. Despite the limited number of metabolomic studies on P-EVs, advancing our understanding of their biochemical contents could pave the way for novel nanomedicines with significant therapeutic potential.

**TABLE 1:** Secondary metabolites or active ingredients of plants and their different therapeutic purposes.

Source	Active constituents	Therapeutic activity	Refs.
Grapefruit	Terpenes, polysaccharides Myo-inositol, quininic acid, aucubin, doconexent, naringin and naringenin	Anti-inflammatory Antioxidation Regenerative	PMID: 37859568 PMID: 37107317 PMID: 33977960
Ginger	Gingerol, shogaol	Anticancer Anti-inflammatory Antioxidation	PMID: 37865333 PMID: 27318094 PMID: 27318094
Ginseng	Ginsenoside Rg3, steroidal saponins, protopanaxadiols, protopanaxatriols, miRNA	Anticancer Regenerative	PMID: 37542285 PMID: 34586821
Garlic	Allicin, ajoenes, quercetin, vinyldithiins, sulfide, mercaptan	Anti‐inflammatory Anticancer Antioxidation	PMID: 32954162 PMID: 34285262
Tea leaf/flower	Gallic acid, caffeine, epigallocatechin gallate, quercetin	Anticancer Anti-inflammatory Antioxidation	PMID: 35256954 PMID: 34656857
Broccoli	Sulforaphane, glucobrassicin, glucoraphanin	Anti‐inflammatory Anticancer Antioxidation	PMID: 28274798 PMID: 35999223
Aloe vera	Aloe-emodin, aloesin β-sitosterol	Assist in anticancer (deliver indocyanine green and doxorubicin) Regenerative, Anti-inflammatory	PMID: 34930289 PMID: 35675505 PMID:36145653
Bitter melon	α-eleostearic acid, cucurbitane triterpenoids, momordicine, kuguaglycoside	Anticancer Anti-inflammatory	PMID: 34454534 PMID: 20179194
Moringa oleifera	miRNAs	Anticancer Antioxidation	PMID: 32550010 PMID: 26930203
Kaempferia parviflora	5,7-dimethoxyflavone, 5,7,4′-trimethoxyflavone	Anticancer	PMID: 35077499 PMID: 30273054
Dandelion	Taraxasterol, taraxerol, caffeic acid, chicoric acid, chlorogenic acid, luteolin	Anticancer	PMID: 33716082 PMID: 35405251
Artemisia annua	Artemisinin, dihydroartemisinin, artesunate, artemether, arteether	Anticancer	PMID: 36879291
Asparagus cochinchinensis	Asparagus polysaccharide, furostanol saponin	Anticancer, Antioxidation	PMID: 31239858; PMID: 22076757

## Pharmacological activity of P-EVs in anticancer therapy

In recent years, the exploration of exosome-based therapeutic strategies has gained significant attention in clinical cancer research. One of the primary goals in modern oncology is to develop targeted tumor therapies that selectively eliminate cancer cells without harming normal tissues. P-EVs have been shown to regulate diverse biological processes and offer potential advantages in cancer treatment (Raimondo et al., 2019). Some studies have investigated the direct encapsulation of pharmacological agents within P-EVs via phagocytosis or endocytosis, facilitating targeted uptake by recipient cells (Wang et al., 2014). Additionally, exosomes derived from traditional Chinese medicine have emerged as promising candidates for disease treatment. For example, Forsythiaside A-loaded, hyaluronic acid (HA)-modified milk-derived exosomes (mExo) have been devised for the inhibition of NLRP3-mediated pyroptosis, offering a novel therapeutic approach for liver fibrosis (Gong et al., 2023). However, a growing area of research focuses on extracting exosome-like nanotherapeutics from plants and integrating them into conventional treatment strategies, opening new avenues for cancer therapy.

Among the various sources of P-EVs, those derived from ginger have demonstrated remarkable anticancer properties. Engineered ginger-derived EVs (GEVs) can be efficiently internalized by gastric adenocarcinoma cells, leading to dose-dependent inhibition of cell viability (Nemidkanam and Chaichanawongsaroj, 2022). Moreover, GEVs loaded with doxorubicin selectively target colon cancer cells, inducing apoptosis and promoting intestinal repair, thereby serving as a potential therapeutic strategy for inflammatory bowel disease and colitis-related cancers (Zhang et al., 2016). Chen et al. have isolated natural nanocarriers from tea flowers containing high levels of polyphenols, flavonoids, functional proteins, and lipids, which induce mitochondrial damage, amplify reactive oxygen species (ROS), arrest the tumor cell cycle, and promote breast tumor apoptosis while inhibiting lung metastasis (Chen et al., 2022). Similarly, Zu et al. developed tea leaf-derived EVs that suppress inflammatory cytokine expression, enhance macrophage-specific endocytosis, and restore gut microbiota diversity, improving treatment outcomes in colitis-related colon cancer (Zu et al., 2021). Additionally, Stanly et al. determined that grapefruit-derived EVs (GFEVs) downregulate the AKT-ERK signaling axis in various cancer cell lines, leading to cell cycle arrest and apoptosis (Stanly et al., 2020). Further advancements include the development of folic acid (FA)-conjugated GFEVs (GFEV-FAs) for targeted delivery of paclitaxel to colon tumors, enhancing therapeutic efficacy (Wang et al., 2013). Collectively, these findings underscore the significant potential of P-EVs in anticancer therapy.

P-EVs are also being explored as adjuncts to conventional chemotherapy and targeted therapy. For instance, GFEVs have been utilized not only to encapsulate paclitaxel (Wang et al., 2013), but also to deliver doxorubicin via heparin-based nanoparticles, enabling drug penetration across the blood-brain barrier and exerting inhibitory effects on glioma (Niu et al., 2021). Yang et al. found bitter melon-derived EVs (BMEVs) to downregulate NLRP3 expression and clear ROS in oral squamous cell carcinoma (OSCC), reducing OSCC resistance to 5-fluorouracil (5-FU) (Yang et al., 2021). Beyond their direct anticancer effects, P-EVs also mitigate side effects associated with conventional therapies. For example, Cui et al. found that BMEVs protect against myocardial cell damage and fibrosis induced by chest radiotherapy by reducing ROS levels and preserving mitochondrial homeostasis, offering potential protection against radiation-induced heart disease (Cui et al., 2022). Additionally, EVs from dandelion and rhodiola significantly alleviate bleomycin-induced pulmonary fibrosis in mice (Du et al., 2019). Notably, GEVs facilitate controlled doxorubicin release in the acidic tumor microenvironment (TME), limiting systemic toxicity (Luan et al., 2017). These findings highlight the promising role of P-EVs in enhancing therapeutic efficacy while minimizing adverse effects.

With growing research interest, P-EVs are being investigated for their potential applications beyond anticancer therapy, including antifibrotic, antiviral, gut microbiota-regulating, and TME-modulating activities. Table 2 provides an overview of the therapeutic applications of P-EVs from various plant sources in cancer treatment.

**TABLE 2:** The therapeutic applications of P-EVs from various plant sources in cancer treatment.

Exosomes	Cancer type	Mechanisms	Ref.
Ginger ELNs	Breast cancer	Install in ICG targeting tumor cells to mediate lipid peroxidation, ROS and MDA accumulation, increasing the efficacy of light mediated therapy	PMID: 39902066
	Colon cancer	Reduce TNF-α, IL-6 and IL-1β and increase IL-10 and IL-22, preventive effect; carry doxorubicin and induce tumor cell apoptosis	PMID: 27318094
Ginseng ELNs	Cold tumor (colon, breast cancer)	Activate tumor-infiltrated T lymphocytes and reprogram TAMs, enhance immune checkpoint antibody efficacy	PMID: 34450250
	Melanoma	Activate TLR4 expression, resulting in the polarization of M2 to M1 phenotype and produce ROS	PMID: 31775862
	Lung cancer	Inhibit PPP and induce downregulation of TP expression, prevent tumor cell EMT	PMID: 36059624
Grapefruits ELNs	Glioma	Help doxorubicin to bypass the blood-brain barrier to the tumor site	PMID: 33475372
	Colon cancer	Combine with FA and carry PTX, inhibit colon tumor cells	PMID: 23695661
	Melanoma	Make cell cycle arrest at G2/M checkpoint and upregulate of cell cycle inhibitor p21, further inhibit Akt/ERK signaling	PMID: 33371199
Garlic SEVs	Kidney and lung cancer	Upregulation of pro-apoptotic genes such as p53, Bax, Cas3 and Cas9	PMID: 34285262
Asparagus cochinchinensis ELNs	Liver cancer	Upregulation of pro-apoptotic protein expression and trigger activation of caspase-9, induce apoptosis	PMID: 33664572
Bitter melon EVs	Oral squamous cell carcinoma	Downregulate NLRP3 expression and reduce the resistance of 5-FU	PMID: 34454534
Tea flowers/leaves ELNs	Breast cancer	Stimulate ROS amplification, increase intracellular ROS, trigger mitochondrial damage and arrest cell cycle, resulting in anti-proliferation, anti-migration, and anti-invasion activities	PMID: 35256954 PMID: 36600299
Moringa oleifera ELNs	Hematologic malignancy	Decrease in B-cell lymphoma 2 protein expression and reduce mitochondrial membrane potential	PMID: 32550010
Brucea javanica ELNs	Breast cancer	Deliver the functional miRNAs to tumor cells, regulate the PI3K/Akt/mTOR pathway and promote ROS/caspase-mediated apoptosis	PMID: 38295998
Citrus limon EVs	Breast cancer	Combine DOX to reduce cell viability and increase ROS content, promote human fibroblast proliferation	PMID: 39481327
Platycodon grandiflorum EVs	Breast cancer	Increase production of ROS, facilitate the polarization of TAMs toward M1 and increase the secretion of pro-inflammatory cytokines	PMID: 39920791

ELNs, Exosome-Like Nanovesicles; FA, folic acid; PTX, paclitaxel; ICG, indocyanine green; ROS, reactive oxygen species; 5-Fu, 5-fluorouracil; TAMs, tumor-associated macrophages; TLR4, Toll-like receptor4; PPP, pentose phosphate pathway; TP, thymidine phosphorylase; EMT, epithelial-mesenchymal transition; DOX, doxorubicin; M1, classically activated macrophages.

## Indirect roles of P-EVs in reshaping the TME

The TME comprises a heterogeneous network of cells, such as infiltrating cancer cells, endothelial cells, epithelial cells, fibroblasts, and mesenchymal macrophages (de Visser and Joyce, 2023). These cells, together with their surrounding extracellular matrix, contribute to the formation of resistant and dynamic “cold tumors,” which evade immune detection and facilitate tumor progression. Excessive secretion of cytokines and chemokines within the TME induces an acidic milieu that sustains proliferative signaling, promotes angiogenesis, enhances invasion and metastasis, triggers pro-tumor inflammation, and enables immune evasion (Xiao and Yu, 2021). Consequently, a major research focus has been the modulation of the TME to transform it from a tumor-supportive to a tumor-suppressive environment. P-EVs have demonstrated significant potential in exerting anti-inflammatory effects and enhancing immune responses within the TME.

Recent studies highlight the potential of ginseng-derived EVs to penetrate the blood-brain barrier via multiple endocytic pathways, where they suppress regulatory T cell (Treg) activation and M2 macrophage polarization within the TME. These EVs downregulate key oncogenic factors, including iNOS, VEGF, EGF, and STAT3, leading to enhanced immune responses and glioma suppression (Kim et al., 2023). In another study, ginseng-derived EVs reprogram tumor-associated macrophages (TAMs), increasing the secretion of CCL5 and CXCL9 to recruit CD8^+^ T cells into the tumor core, thereby enhancing the efficacy of immune checkpoint blockade therapy with PD-1 monoclonal antibodies (Han et al., 2022). These findings further validate the immunomodulatory potential of ginseng, reinforcing its designation as the “king of herbs.”

Within the TME, the cGAS-STING pathway plays a pivotal role in modulating immune and inflammatory responses. Liu et al. successfully isolated and purified nanoscale vesicles from Artemisia annua and demonstrated that these vesicles can reprogram tumor-associated macrophages (TAMs) from a pro-tumor phenotype to a pro-inflammatory state. This transformation activates the cGAS-STING pathway, further reprogramming macrophages and enhancing cytotoxic T-cell responses, ultimately contributing to tumor regression and improving the efficacy of αPD-L1-mediated immunotherapy in lung cancer (Liu et al., 2023). Additionally, *Dendropanax morbiferus*-derived P-EVs have been reported to inhibit cancer-associated fibroblasts (CAFs) by modulating gene expression related to growth factors or extracellular matrix components including integrins and collagen. These findings highlight the potential of P-EVs as future anti-CAF agents (Kim et al., 2020). Autophagy, a self-protective process responsible for preserving cellular homeostasis, enables cancer cells to survive under nutrient-deficient and hypoxic conditions within the TME (Vessoni et al., 2013). Exosome-like nanovesicles from *Brucea javanica* have been found to suppress autophagy through the promotion of PI3K/Akt/mTOR phosphorylation while inducing apoptosis via ROS/Caspase activation and inhibiting the JAK/STAT signaling pathway, thereby exerting potent anti-cancer effects (Yan et al., 2024; Chen et al., 2020; Wang et al., 2019).

**FIGURE 2:**
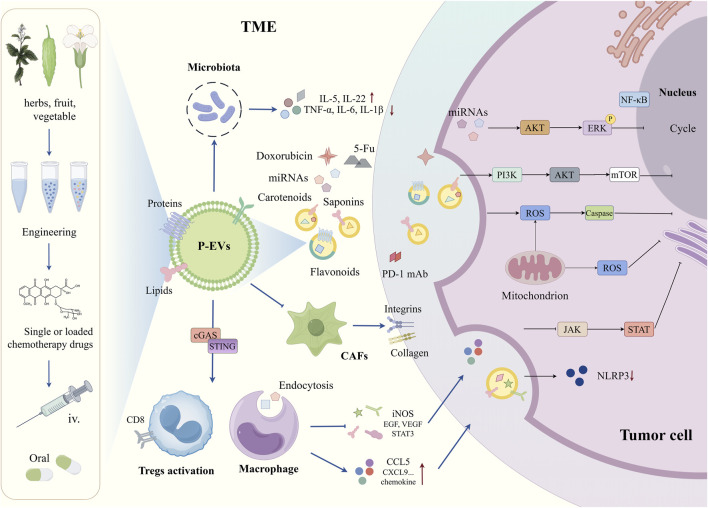
The existing pathways cascade of P-EVs in the TME (partial). TME, tumor microenvironment; CAFs, cancer-associated fibroblasts; Tregs, regulatory T cells; iv, intravenous; 5-Fu, 5-Fluorouracil; cGAS, cyclic GMP-AMP synthase; STING, stimulator of interferon genes; iNOS, inducible nitric oxide synthase; EGF, epidermal growth factor; VEGF, vascular endothelial growth factor; STAT, signal transducer and activator of transcription; CCL5, C-C motif chemokine ligand5; CXCL9, C-X-C motif chemokine ligand9; PD-1 mAb, programmed cell death protein1 monoclonal antibody; NF-kB, nuclear factor kappa-light-chain-enhancer of activated B cells; AKT, protein kinase B; ERK, extracellular signal-regulated kinase; PI3K, phosphatidylinositol 3-kinase; mTOR, mechanistic target of rapamycin; ROS, reactive oxygen; JAK, janus kinase; NLRP3, NOD-like receptor family, pyrin domain containing3.

Inflammation is a fundamental and dynamic component of the TME, closely linked to metastasis, drug resistance, and poor prognosis (Jiang et al., 2025). As previously mentioned, GEVs can repair intestinal mucosal damage and prevent colitis-associated tumor formation. The underlying mechanism involves the upregulation of anti-inflammatory factors such as IL-10 and IL-22 and the downregulation of pro-inflammatory cytokines, including TNF-α, IL-6, and IL-1β, thereby modulating the inflammatory cytokine profile within the TME (Zhang et al., 2016). Numerous studies have emphasized the roles of miRNA-146a and miRNA-125a in negatively regulating the NF-κB pathway (Meisgen et al., 2014; Curtale et al., 2019), which is closely associated with macrophage stimulation, pro-inflammatory cytokine production, and chemokine release (Dorrington and Fraser, 2019). Notably, exosome-like vesicles derived from apples have been shown to induce the transcriptional activation of miRNA-146a and miRNA-125a, leading to a reduction in IL-8 and IL-1β expression, suppression of JNK and NF-κB pathway activation, and overall modulation of the inflammatory TME (Trentini et al., 2022). Collectively, these findings suggest that P-EVs possess the potential to transform “cold tumors,” which are typically resistant to conventional therapies, into “hot tumors” that exhibit enhanced responsiveness to treatment, thereby significantly improving therapeutic outcomes (Figure 2).

Overall, recent studies underscore the promising anti-cancer potential of P-EVs in targeting the TME. However, the TME comprises a vast and intricate network of cellular interactions, making it far more complex than initially perceived. In addition to cancer cells, other cell types secrete extracellular vesicles, and factors such as hypoxia, low pH, and nutrient scarcity pose additional challenges for P-EV research. Nevertheless, the emergence of P-EVs offers a promising avenue for the development of low-toxicity cancer therapies.

## Discussion

Targeted cancer therapy is expected to remain a sustainable and advancing field of research. Engineered extracellular vesicles derived from autologous cells or mammalian sources face challenges such as immune rejection, toxicity, and limited clearance rates. In contrast, P-EVs represent a relatively novel alternative with advantages including natural origin, low toxicity, reduced immunogenicity, and excellent biocompatibility (Den et al., 2017). Over the past decade, various anti-tumor mechanisms of P-EVs have been explored, with most studies focusing on their role in inducing cancer cell apoptosis (Stanly et al., 2020; Yang et al., 2021; Yan et al., 2024). However, research on the effects of P-EVs on the TME and its associated immune cells remains in its infancy. Compared with P-EVs, engineered mammalian cell-derived EVs are more flexible in cancer therapy. For example, endothelial cells derived from bone marrow derived mesenchymal stem cells (BMSCs) have the advantages of biocompatibility and low immunogenicity. Previous studies have expressed streptavidin in the cellular membrane and EVs derived from MSCs, which was engineered into plasma membrane-located proteins its fusion with the coding sequence of signal peptides and transmembrane regions, to achieve the functionalization of BMSC-EVs (Meng et al., 2023). It is worth mentioning that immune cells, such as natural killer cells, can also secrete EVs (NKEVs) with tumor-targeting capabilities. It has been reported that it can encapsulate paclitaxel through electroporation and target breast cancer cells to exert inhibitory effects (Han et al., 2020). However, NKEVs contain many molecules related to immune responses, including IFN-γ, TNF-α, IL-10, MHC-I and MHC-2, and other chemokines, etc. This means that for engineered NKEVs, rejection reactions must be taken into account (Wu et al., 2021). In contrast, P-EVs do not include the above-mentioned cytokines or chemokines, low human homology and its composition is simpler than that of mammalian cell-derived EVs (Wang et al., 2014). Furthermore, Zhou et al. have engineered BMSCs derived from autologous stem cells, loaded the obtained EVs with miR-138-5p and the anti-fibrotic agent pirfenidone (PFD) and subjected to surface modification with integrin α5-targeting peptides to reprogram cancer-associated fibroblasts (CAFs) and enhance its tumor-targeting ability, remodeling the TME of pancreatic cancer, improving tumor hypoxia and enhancing gemcitabine sensitivity (Zhou et al., 2024). Mammalian derived EVs can rely on surface proteins (such as integrins) to achieve natural targeting ability, while P-EVs has not been fully developed the natural targeting property and lacked protein markers with biochemical characteristics. Currently, commonly measured markers include CD63, CD81and CD9, etc. (Hong et al., 2023; Jokhio et al., 2024), the specific biomarkers of P-EVs are still lacking, which required more engineering modification. Overall, compared to mammalian cell-derived extracellular vesicles, additional studies are required to better understand the biogenesis, protein surface markers, and targeting mechanisms of P-EVs.

In addition to low immunogenicity, high biocompatibility and low toxicity are also advantages of P-EVs. For example, sodium thiosulfate (STS) is a clinically approved drug for the treatment of vascular calcification, but its efficacy is limited by poor bioavailability and severe adverse effects. Feng et al. have created a bionic GFEVs that can be loaded with STS and targeted delivery without inducing hemolysis or causing any damage to other organs (Feng et al., 2023). Some edible fruit plants are non-toxic by themselves and can prevent the toxic and side effects brought about by traditional treatments. For instance, grapes derived EVs can effectively prevent oral mucositis in patients with head and neck tumors caused by radiotherapy and chemotherapy. A Phase I clinical trial has now been initiated (NCT01668849). Fang et al. have isolated and purified kiwifruit derived EVs (KEVs) and delivered the lipophilic multi-targeted kinase inhibitor sorafenib (KEV-SFB) in a targeted manner, evidence indicates that the KEV-SFB reduces the leakage of SFB in the gastrointestinal environment, which was able to achieve liver accumulation and was predominantly taken up by HepG2 cells in mice without toxic effects on normal hepatocytes (Fang et al., 2023). Previous researchers have successfully loaded doxorubicin in GEVs (DOX-GEVs) and released DOX into the acidic TME in a pH-dependent manner, which has been proven to be effectively absorbed by colon-26 and HT-29 through endocytosis and has non-toxic *in vivo* (Zhang et al., 2016). Despite P-EVs have general low toxicity, they are not entirely free from adverse effects. For instance, intravenous administration of exosome-like vesicles derived from tea flowers has been shown to induce tumor cell cycle arrest and apoptosis. However, mice receiving intravenous treatment exhibited significant weight loss and signs of liver toxicity compared to those treated via oral administration (Chen et al., 2022). This finding suggests that oral administration may be a safer and more viable route for P-EV delivery. Nevertheless, enzymatic degradation in the digestive tract, interactions with gut microbiota, and external factors such as diet and concomitant drug administration can influence orally administered engineered P-EV bioavailability and therapeutic efficacy. Thus, there is an urgent need to develop novel P-EV-based treatment strategies that are non-toxic, stable, and readily absorbable while maintaining compatibility with other therapeutic agents. Ensuring the efficient release and targeted delivery of P-EVs following systemic administration remains a critical challenge.

Addressing the complexities of the ever-evolving TME has been a formidable challenge in recent years. Tumor-derived exosomes (TDEs) carry various bioactive molecules, including TGF-β, caveolin-1, HIF-1α, and β-catenin, which collectively enhance the invasive and migratory capabilities of recipient cells, promote immune evasion, and contribute to drug resistance (Syn et al., 2016). Although engineered P-EVs can cross biological the blood-brain barrier and skin barrier, their precise route of delivery to tumor target cells within the TME remains highly complex and challenging. At present, most studies focus on developing drug delivery platforms for engineered EVs, while very few studies concentrate on how to enhance the cancer cell and tumor-targeting specificity. Chemical modification of the surface of P-EVs may improve targeting ability and reduce the off-targeted side effects. There is a study has modified GEVs using the tumor-targeting ligand iRGD, iRGD is a cyclic peptide widely used in tumor-targeted drug delivery and has a high binding affinity for integrins and NRP-1 receptors that are overexpressed in tumors. Compared with unmodified GEVs, GEV-iRGD significantly enhances the ability to be absorbed by tumor cells (Wang et al., 2025). Kang et al. have enhanced the targeting of red cabbage-derived EVs (RabEVs) to intestinal epithelial cells and immune cells by coupling hyaluronic acid (t-Rabex) with the surface of RabEVs, thereby highlighted their anti-inflammatory, antioxidative, and tight-junction maintenance properties in inflammatory bowel disease (IBD) (Kang et al., 2024). In a recent study, Yang et al. have integrated the cell membrane fragments of breast cancer cell line 4T1 into lemon-derived nanovesicles (LEVs), and then loaded the anti-cancer drug DOX to establish the composite nanomedicine delivery vector LEVBD, which significantly improved the targeting of homologous tumors and promoted the transcellular transport of drugs, suppressed tumor growth in mice after intravenous injection, and had no observable toxic and side effects (Yang et al., 2025). It can be seen from these studies that not only the development of P-EVs has aroused the interest of researchers, but also how to better transform these natural EVs to make them more targeted and penetrating is the direction that more and more scholars are curious about and focus on. However, the stability of P-EVs is uncertain. For example, Temperature and pH can affect its integrity and activity. Zhang et al. once used a simulated gastrointestinal fluid system to evaluate the particle size and zeta potential changes of GEVs, the results showed that compared with the buffer PBS, the size of GEVs increased in the stomach-like solution and further enlarged in a small intestine like solution (Zhang et al., 2016). This is also the risk and challenge faced by oral administration in exerting its efficacy. It further underscores the necessity of enhancing P-EV stability. Future research on P-EVs should prioritize improving their purity and yield, optimizing cost-effectiveness during isolation and storage, and ensuring their safety and efficacy for clinical applications. Efforts should be directed toward accelerating early-stage clinical investigations to bring P-EVs closer to therapeutic implementation. With continued advancements, P-EVs hold significant promise for revolutionizing cancer treatment by providing a natural, effective, and low-toxicity therapeutic approach.
